# Bioactives from Bee Products and Accompanying Extracellular Vesicles as Novel Bioactive Components for Wound Healing

**DOI:** 10.3390/molecules26123770

**Published:** 2021-06-21

**Authors:** Željka Peršurić, Sandra Kraljević Pavelić

**Affiliations:** 1 Faculty of Chemical Engineering and Technology, University of Zagreb, Marulićev trg 19, HR-10000 Zagreb, Croatia; persuric@fkit.hr; 2 Faculty of Medicine, Juraj Dobrila University of Pula, Zagrebačka 30, HR-52100 Pula, Croatia; 3 Faculty of Health Studies, University of Rijeka, Viktora Cara Emina 5, HR-51000 Rijeka, Croatia

**Keywords:** bioactive compounds, bee products, extracellular vesicles, wound healing, antimicrobial, antioxidant

## Abstract

In recent years, interest has surged among researchers to determine compounds from bee products such as honey, royal jelly, propolis and bee pollen, which are beneficial to human health. Mass spectrometry techniques have shown that bee products contain a number of proven health-promoting compounds but also revealed rather high diversity in the chemical composition of bee products depending on several factors, such as for example botanical sources and geographical origin. In the present paper, we present recent scientific advances in the field of major bioactive compounds from bee products and corresponding regenerative properties. We also discuss extracellular vesicles from bee products as a potential novel bioactive nutraceutical component. Extracellular vesicles are cell-derived membranous structures that show promising potential in various therapeutic areas. It has been extensively reported that the use of vesicles, which are naturally formed in plant and animal cells, as delivery agents have many advantages. Whether the use of extracellular vesicles from bee products represents a new solution for wound healing remains still to be elucidated. However, promising results in specific applications of the bee products in wound healing and tissue regenerative properties of extracellular vesicles provide a good rationale to further explore this idea.

## 1. Introduction

Honeybees (*Apis mellifera* L.) produce several valuable products used as health products or food since ancient times. Honey is a substance produced by bees through a process involving the ingestion of flower nectar and subsequent processing known as inversion [1]. Propolis is a sticky mixture produced by mixing saliva and beeswax with exudates collected by honeybees from trees or other botanical sources used to protect the hives from invaders and microorganisms, as well as to provide thermal insulation and reinforce hives by filling cracks and holes [2]. Royal jelly is a form of hypopharyngeal and mandibular gland secretion of nurse bees, used to feed young worker larvae for the first three days and as food for the queen bee throughout her life cycle [3]. Bee pollen is a result of the agglutination of flower pollen with nectar and salivary substances of bees [4]. Bee products are generally considered a high-quality source of bioactive compounds, and it is not surprising that they have gained interest in recent years [5]. Studies have demonstrated that their therapeutic effect is strongly associated with the plant source and bioactive composition [1,4,6,7].

## 2. Bioactive Compounds in Bee Products

Honey is a supersaturated sugar solution containing mainly fructose and glucose. Lower amounts of disaccharides, fructo-oligosaccharides, proteins, organic acids, amino acids, minerals, vitamins and enzymes are also present [3,8]. Among vitamins, honey contains ascorbic acid, vitamin B6, thiamine, riboflavin, niacin and pantothenic acid. Minerals found in honey include calcium, copper, magnesium, iron, manganese, potassium, phosphorus, sodium and zinc [9]. In addition, honey contains valuable bioactive compounds, especially polyphenols and tocopherols. The two main phenolic groups represented in honey are phenolic acids (in particular *p*-coumaric, ellagic, caffeic, and ferulic acids) and flavonoids (including pinocembrin, apigenin, kaempferol, quercetin, chrysin, galangin, and hesperetin). However, the composition of the honey differs according to the plant from which the bees have collected the nectar [1,3,8]. For example, honey produced from *Echium plantagineum* L. has a higher content of phenolic acids than flavonoids and two characteristic phenolic compounds: caffeic and *p*-coumaric acid. In addition, high-performance thin-layer chromatographic (HPTLC) analysis of phenolic compounds in pine, chestnut and sunflower types of honey from Turkey showed that each type of honey had a characteristic HPTLC fingerprint. The main compounds in sunflower honey samples were chlorogenic acid, quercetin and caffeic acid; chestnut honey samples were rich in phenolic acids, while quercetin and caffeic acid were characteristic markers for the pine honey samples [10]. Moreover, the content of these bioactive compounds may vary to a large extent depending upon the geographical origin. For example, in *Rhododendron* honeys obtained from different areas of Turkey (Black Sea Region), the total phenolic content ranged from 0.24 to 141.83 mg of gallic acid equivalents per 100 g of honey [11]. As expected, antioxidant activity determined by the use of the phosphomolybdenum method varied as well between samples from different areas, ranging from 12.76 to 80.80 mg of ascorbic acid equivalents per g (s) of honey. An improved understanding of the phenolic composition of honey samples and the corresponding variability influenced by the plant origin and the apiary location has been achieved in the recent decade by the use of newly developed methods based on high-resolution mass spectrometry (HRMS) [12,13,14]. These methods allow complete profiling of phenolic compounds in different mono- and polyfloral kinds of honey and could therefore be used to assess the authenticity of honey according to its geographical and botanical origin.

Propolis, also known as “bee glue,” is a lipophilic material made from resinous substance, wax, essential oil, pollen and other compounds representing a portion of 5% of the whole propolis content [15]. Interestingly, more than 300 organic compounds were identified in propolis, including vitamins, chalcones, polyphenols and terpenoids [16,17,18]. the two best-described types of propolis that can be distinguished based on plant origin are the European brown propolis type and the Brazilian green propolis [17]. The European type of propolis is obtained from the species *Populus* spp. and is characterized by a high content of flavonoids (including pinocembrin, galangin, chrysin and pinobanksin), phenolic acids and their esters [15]. Metabolomic analysis of the *Populus* type propolis with various spectroscopic techniques showed that the altitude of propolis collection origin influenced its final chemical composition. In particular, propolis collected above 500 m had a high content of phenolic glycerides originating from the *P. tremula* buds, whereas major bioactive compounds in propolis samples collected below 400 m were flavonoids originating from *P. nigra* and *P. x euramericana* buds. Propolis samples collected between 400 and 500 m had variable amounts of all detected metabolites and could not be associated with a single origin [19]. Besides, a new type of propolis, the Mediterranean type, was identified in Europe based on the metabolomic profile. In the Mediterranean propolis type, the predominant components are diterpenes from the plant *Cupressus sempervirens* [20,21]. Our previous research has shown that different types of propolis with different polyphenolic profiles can be found in a relatively small geographical area, indicating the need for a comprehensive chemical characterization of propolis samples before developing pharmaceutical applications [22]. The liquid chromatography-tandem mass spectrometry (LC–MS/MS) analysis of potential allergens in various propolis extracts confirmed additionally the need for a deeper evaluation of the propolis polyphenolic composition as new hydroxycinnamic acid derivatives with potential allergenic properties were discovered [23]. The polyphenolic composition of propolis particularly determines its antioxidant activity, and even small changes in the final content can influence the pharmaceutical properties. For example, Papachristoforou et al. (2019) analyzed propolis from different areas of a small Greek island Samothraki, known for its high plant biodiversity, and discovered a huge variability in the antioxidant activity depending on the territory and the local flora [24]. In particular, the sample with the lowest antioxidant activity had only 1.75 μmol of Trolox equivalents per gram of dry weight (TRE/g), while in the sample from the other area, a maximum value of 1813.2 μmol TRE/g was measured.

Royal jelly is a yellowish-white and viscous jelly-like substance that contains mainly water, proteins and carbohydrates with a small percent of lipids [3]. The minor components of royal jelly are minerals (Fe, Na, Ca, Mg, K, Zn, Mn and Cu), amino acids, vitamins (A, B complex, E and C), enzymes, polyphenols, hormones, nucleotides and minor heterocyclic compounds [7]. The most important protein in royal jelly is royalactin, or the major royal jelly protein 1 (MRJP1), known for its impact on the modulation of biological functions in a broad range of species [25]. Quantitative analysis of fresh royal jelly by using LC–MS/MS showed that the content of MRJP1 in royal jelly varies from 41.96 to 55.01 mg/g [26]. Besides MRJP1, other bioactive compounds present in royal jelly are 10-hydroxy-2-decenoic acid (HAD), adenosine monophosphate (AMP) N1 oxide, adenosine, acetylcholine and polyphenols [3,7,27]. Analysis of polyphenols in royal jelly samples by turbulent flow chromatography coupled with liquid chromatography–Orbitrap mass spectrometry revealed the highest concentrations of phenolic acids followed by compounds from the groups of flavanones, flavones, flavonols and isoflavonoids [28].

Bee pollen is made after the foragers collect the pollen and the bees store it into the cells of the brood comb with a small amount of honey to prevent spoilage and maintain high quality. The stored pollen that undergoes chemical processes and changes, such as the natural lactic acid fermentation process, is known as bee bread [29,30]. Bee pollen contains ingredients with high biological value. It is made of essential amino acids, essential fatty acids, vitamins, minerals and polyphenols [5,31,32]. Bee pollen is often referred to as the “perfectly complete food,” as it contains all essential amino acids needed for humans [4]. Among minerals, bee pollen is rich in health-promoting macroelements Ca, K and Mg and microelements Cu, Fe, Mn and Zn, amongst which Ca and Mg showed the highest bio-accessibility [33]. Bioactive characteristics of pollen are significantly defined by the composition of phenolic compounds in the bee pollen. The main groups of phenolic compounds in the bee pollen are phenolic acids and flavonoids [34]. Accordingly, the analysis of bee pollen and bee bread samples from Turkey using LC–MS/MS revealed the presence of 23 phenolic compounds and 42 free amino acids [35]. Among phenolic compounds, rutin had the highest share in both samples, whereas the phenolic compounds 2,5-dihydroxybenzoic acid, protocatechuic acid and kaempferol had higher occurrence rates in bee bread than in bee pollen. The phenolic profile did not solely depend on the product type, but differences were rather noticed due to the sample region-associated impact as well. The free amino acid profile showed a dominance of proline that can be also used as a parameter of sample freshness assessment. Samples also had a high content of L-asparagine and L-aspartic acid. HPTLC fingerprinting of bee pollen from different regions of Serbia conducted by Mosić et al. (2019) showed that the phenolic composition of pollens is also under botanical origin influence [36]. The bee pollen samples had a total phenolic content (TPC) ranging from 5.60 to 30.24 mg of gallic acid per gram of pollen, the lowest value being found in the sample with a predominance of the *Fabaceae* pollen type and the highest value in the sample with the greatest dominance of rust spores. Furthermore, the authors determined the qualitative profile of flavonoid glycosides present in bee pollen samples using ultra-high-performance liquid chromatography (UHPLC) coupled with a linear ion trap–Orbitrap mass spectrometry (UHPLC–LTQ Orbitrap MS) technique. All identified compounds were from the same group of flavonols, i.e., 3-*O*-glycosides of quercetin, isorhamnetin and kaempferol. The highest number of flavonol glycosides was identified in the sample with a predominance of the *Rosaceae* pollen type. The variability of TPC, flavonoid content and antioxidant activity was also noticed among honeybee pollen samples from the central zone of Chile of different botanical origin [37]. For example, values of antioxidant capacity, evaluated by ferric reducing antioxidant power, were between 19 ± 2 and 194 ± 7 µmol TRE/g bee pollen.

**Figure 1:**
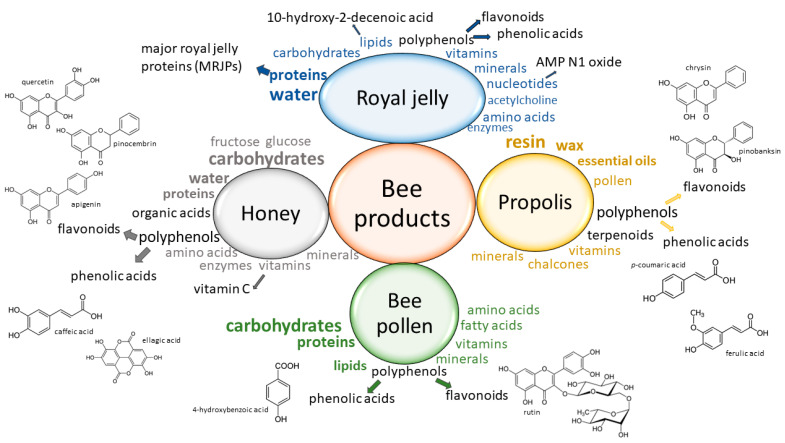
The chemical composition of bee products with emphasis on the main compounds (depicted in black font letters) responsible for the antimicrobial, anti-inflammatory and/or antioxidant activity of bee products. Examples of phenolic acids and flavonoids chemical structures in bee products are presented left and right accordingly [3,16,39,40].

Previous studies showed that bee products (honey, royal jelly, propolis and bee pollen) had a rich and diverse composition of bioactive compounds with beneficial effects that require dedicated characterization and research (Figure 1). Many factors, such as the botanical source, decisively influence the bioactive composition of the bee products and impart specific biological properties [38]. The huge variability in the chemical composition of the bee products complicates the determination of authenticity parameters, as well as the determination of the corresponding biological activity. Therefore, complete elucidation of the bioactive composition of bee products is essential for a more comprehensive understanding of their role in modern medicine. Certainly, HRMS methods will have a great impact on the complete chemical profiling of bee products.

### Bioavailability of Bioactive Compounds from Bee Products

The effect of bee products on the human body is strictly dependent on the bioavailability of the phytochemicals and other bioactive compounds as well as their metabolism. Apart from the bee product composition, other factors are involved in the absorption and metabolism of nutrients and bioactive compounds, particularly phenolic components, from the ingested bee products. These factors include interaction with other compounds from the bee matrix or from the food, the chemical structure of the phenolic compounds and intestinal factors, including the microbiota status. Just as an example, flavonoids bioavailability from honey was studied by Schramm et al. (2003), who demonstrated increased total plasma phenolic content in human subjects after honey consumption [41]. However, it is generally accepted that each phenolic group has its own absorption and metabolic pattern that underlies biological effects. In the first phase, hydrolysis of flavonoids occurs under intestinal and bacterial enzyme activity. Released aglycones enter directly into epithelial cells, whereas polar glucosides are transported into epithelial cells through an Na-dependent glucose transporter 1 (SGLT1), where they are further subjected to hydrolysis. Before entering the bloodstream, the second phase of flavonoid metabolism occurs, in which conjugated products are formed. The third phase of metabolism involves proteins associated with multi-resistance (MRP1, MRP2). Some flavonoids are resistant to hydrolysis and pass directly to the colon, where the resident microbiota performs biotransformation into the products available to the liver within the enterohepatic circulation. Other metabolites, however, after the metabolic changes taking place in hepatocytes, are secreted by some organic acid transporters in the systemic circulation, and they are either absorbed by cells or tissues or excreted by the kidneys [1]. Another example is the bioavailability of bioactive compounds from bee pollen is rather limited, which could be attributed to the complex structure of the pollen grain wall and the high resistance of the outer layer to biodegradation. The outer layer has a biopolymer called sporopollenin that is resistant to the action of digestive enzymes, and only animals, which developed mechanisms for extraction of bioactive compounds from pollen grains, can exploit more than 50% of the pollen grain content [31]. Various methods and processes have been proposed to enhance the bioavailability of phytonutrients from bee products. Enhanced bioavailability and controlled release of phenol compounds within the intestine may be obtained, for example, by use of nanocarriers (i.e., encapsulation in biopolymer-based technologies or nano-encapsulation by natural nano-carriers), prodrugs (chemical modification of bioactive compounds) and phytosomes (lipid-molecule complexes) [42].

## 3. Health Benefits of Bee Products with Focus on Regenerative Medicine

The health benefits of bee products have been studied extensively. Some of the observed beneficial health effects include documented anti-inflammatory, antibacterial, antifungal, antiviral and antioxidant effects. Some honeybee products also showed antitumor effects [43,44,45]. The majority of the research has been done in preclinical setup, and the observed health effects are mainly attributed, but not confined entirely, to polyphenolic constituents. This is because the mechanisms of action are complex and involve a wide range of molecular processes that are important in the pathogenesis of many diseases. Clinical evidence supports the usefulness of honey in wound healing, treatment of diabetes mellitus and diarrhea [46]. In addition, clinical studies with propolis have shown that propolis is well-tolerated and non-toxic in vivo [47], lowers concentrations of free radicals in wounds [48], exerts antidiabetic effects [49] and improves oral health in chemotherapy-induced oral mucositis [50]. More recently, a clinical trial has been initiated to assess the propolis supplementation effect in patients with coronavirus (COVID-19) [51]. Herein, we will focus on the regenerative properties of bee products due to the combined antimicrobial, antioxidant and anti-inflammatory properties at the molecular level, which contribute to cell and tissue homeostasis and consequently to the regeneration of damaged tissue.

### 3.1. Wound Healing and Tissue Regeneration

Honey has been considered since ancient times as a remedy that can promote the healing of wounds. Egyptians, Greeks, Romans and Chinese used honey to heal wounds and diseases of the gut [3]. The wound-healing effect of honey and other bee products is most likely associated with documented antimicrobial activity and free radicals counteracting. The antimicrobial properties of honey are based on two main mechanisms: inhibition of microbial growth by hydrogen peroxide and through non-peroxide activities. Hydrogen peroxide is produced from glucose by the enzymatic activity of glucose oxidase and has bacteriostatic and DNA-degrading activities on the bacteria. Non-peroxide activities rely mainly on the action of complex phenolic compounds and organic acids. The composition of phenolic compounds and organic acids in honey depends on the floral source collected by bees, and therefore, not all honey types have non-peroxide activities [52,53]. For example, non-peroxide activity in manuka honey, known as the unique manuka factor, is associated with compound methylglyoxal that is present in high levels in manuka nectar [54,55]. The evaluation of mono-floral *Agastache* honey has revealed that this type of honey possesses both mechanisms of antimicrobial activity and is effective against skin infection caused by staphylococci and *P. aeruginosa*. Furthermore, LC-MS/MS analysis uncovered phenolic compounds phenyllactic acid, 4-hydroxybenzoic acid, *p*-coumaric acid and gallic acid, which are known to have antimicrobial activity. In particular, the authors suggested further investigation of methyl syringate as this compound was present in high amounts in *Agastache* honey [56]. In addition to two mechanisms, the antimicrobial activity of honey has been attributed to a combination of different honey characteristics, such as low pH and high osmolarity and to the presence of specific volatile compounds or antimicrobial agent lysozyme [53,57,58].

Besides the antimicrobial activity, the wound healing effect is associated with the viscosity of honey as it allows the creation of a protective barrier that prevents infections in the wounds. The high sugar content also contributes to healing by improving the local nutrition of the damaged areas [53]. The immunomodulatory activity of honey is another biological activity crucial for the wound healing process. Interestingly, depending on the wound microenvironment, honey can both promote and suppress the inflammatory process by stimulating or inhibiting the release of certain cytokines or by activation or reduction of reactive oxygen species levels by neutrophils. Furthermore, honey enhances the activity of cells that are important for the wound closure process, such as human keratinocytes, fibroblasts and endothelial cells [59,60].

Propolis showed similar mechanisms involved in the wound healing process, including antimicrobial, anti-inflammatory and antioxidant activity, as well as giving support to the healing process [21,61,62]. The beneficial role of propolis on wound healing is mainly attributed to its antimicrobial activity, especially to the ability to inhibit biofilm generation [63]. Wojtyczka et al. (2013) showed that ethanolic extract of propolis was effective against methicillin-sensitive *Staphylococcus aureus* (MSSA) and methicillin-resistant *S. aureus* clinical isolates [64]. Likewise, the antimicrobial activity of propolis against *S. aureus* and *Listeria monocytogenes* was confirmed in the study with Serbian propolis [65].

Similarly, propolis has shown both immunosuppressive and immunostimulant effects as well the ability to increase the wound repair abilities of cells that are important for the wound closure process, such as in human keratinocytes. Martinotti et al. (2019) demonstrated that propolis stimulated keratinocytes to close the wound and identified H_2_O_2_ as the main mediator of propolis regenerative properties [66]. Even though propolis has strong antioxidant activity, it may act as a prooxidant and oxidative stress inductor as it stimulates the production of low concentrations of reactive oxygen species such as H_2_O_2_ under certain circumstances. This extracellularly released H_2_O_2_ then traverses the plasma membrane through water- and glycerol-transporting protein aquaporin-3 that modulates intracellular responses. Additionally, in burned wound tissue, propolis stimulated glycosaminoglycan synthesis and release, necessary for granulation tissue formation during the wound healing process. Moreover, it accelerated chondroitin/dermatan sulphates structure modification responsible for the binding of growth factors [67]. It is also important to mention the antioxidant activity of propolis, which is also responsible for the wound healing properties of propolis. Compounds directly associated with the observed antimicrobial, antioxidant, prooxidant and consequently to the wound healing properties are flavonoids and phenolic acids. However, due to high variability in phenolic profiles of propolis, quality control methods for extensive characterization of chemical constituents and pharmacological properties of propolis are required to improve the clinical use of propolis in wound healing [68].

Several research groups assessed combinations of different bee products and their eventual synergistic effect in wound healing and quality and speed of healing. The combined application of propolis and honey was, accordingly, very effective in wound healing of rat skin, which was attributed to a synergistic effect between propolis and honey [69]. Nevertheless, these results were not confirmed in the in vitro study. Ebadi et al. (2021) tested the simultaneous application of propolis and honey for wound healing under in vitro conditions and showed that both products efficiently facilitate migration, proliferation, and viability of human dermal fibroblast cells in a dose-dependent manner. However, in this study. the synergistic effect of propolis and honey was not observed [2].

Royal jelly is another bee product with wound healing properties that seems to play a significant role in wound healing [7,70]. Lin et al. (2020) identified proteins as components important for the wound healing bioactivity of royal jelly. Major royal jelly proteins (MRJP2, MRJP3 and/or MRJP7) enhanced the proliferation and migration of human epidermal keratinocytes in in vitro scratch wound model. Furthermore, these proteins were proposed as valuable lead compounds for the development of novel wound healing medications [71]. Additionally, the same research group tested in vivo and in vitro wound-healing effects of royal jelly collected during the blossom seasons of *Castanea mollissima* Bl. and *Brassica napus* L. [72]. Hydrophilic and lipophilic extracts from *C. mollissima* Bl. royal jelly promoted proliferation and migration of keratinocyte, which accelerated re-epithelialization, while the same fractions from *Brassica napus* L. royal jelly were deprived of such effects. Extracts from both royal jellies showed anti-inflammatory activity by suppressing the production of nitric oxide (NO). However, only the hydrophilic extracts from *B. napus* L. royal jelly could also repress LPS-stimulated production of crucial pro-inflammatory cytokines tumor necrosis factor-α (TNF-α). In addition, hydrophilic extracts from both royal jellies enhanced the production of growth factors important for cell proliferation. The authors concluded that *C. mollissima* Bl. royal jelly has stronger wound healing potential, and the hydrophilic extracts of both royal jellies have a stronger anti-inflammatory effect due to the water-soluble bioactive substances. Royal jelly was suggested as a possible effective therapy not just for skin injuries but also for open sores that develop on the inside lining of the stomach. Specifically, royal jelly showed high healing potential for acetic-acid-induced gastric ulcers in rats [73].

Royal jelly, propolis and honey showed promising effects in the treatment of chronic wounds, especially diabetic foot injuries [60,74,75,76]. The wound healing process in diabetic patients is very demanding as persistent inflammation due to different factors can lead to the chronicity of the wounds [77]. In a randomized placebo-controlled study, the effect of propolis as an adjuvant in the healing of human diabetic foot ulcers was assessed [74]. Treatment of diabetic foot wounds with propolis promoted a reduction in the wound’s area related to an increase in the connective tissue deposit, increased glutathione (GSH) and the GSH/glutathione disulfide ratio, depleted TNF-α and increased interleukin-10 levels. In another randomized controlled study, the administration of topical propolis ointment in addition to the conventional treatments of diabetic foot ulcers reduced the size of ulcers, especially at the beginning of the application [75]. Topical application of bacterial cellulose membrane associated with red propolis accelerated the wound healing process in a diabetic mouse model [76]. This was achieved by controlling prolonged inflammation, reducing the size of the lesion, promoting epithelization and increasing TGF-β levels. The compounds which are considered responsible for these effects are flavonoids.

Flavonoids have also been recognized as an active constituent of bee pollen in the treatment of burns. Flavonoids from bee pollen, such as kaempferol and quercetin, showed anti-edematous, anti-inflammatory and analgesic activity [32].

### 3.2. New Possibilities to Enhance Effect of Bee Products in Wound Healing

Honey and propolis are attractive natural materials for the development of new biomaterials in regenerative medicine and could have a great impact on tissue engineering [53,78,79]. Regenerative medicine is a biomedical field focused on the repair or replacement of damaged tissues and organs by restoring the integrity and functionality of the tissue. Promising results in promoting tissue regeneration have been achieved using scaffolds made of various materials. The materials used for scaffolds are temporary structures to promote cell regeneration that meet specific criteria, particularly desirable mechanical properties, bioactivity, and rate of degradation. Various natural products have been incorporated into scaffolds with the aim of increasing their bioactivity, and bee products have also gained much attention in this field [53].

Tyliszczak et al. (2019), for example, tested the biosafety of chitosan-based hydrogels modified with natural substances caffeine, bee pollen, sage, and *Aloe vera* juice. Hydrogels made from chitosan of bees’ origin did not show cytotoxicity and were characterized by flexibility and diversified surface morphology. Hence, these hydrogels can be considered modern wound dressings that not only absorb wound exudate but contain natural substances with therapeutic properties as well [80]. Furthermore, novel nanofibers made from honey incorporated into poly(1,4-cyclohexane dimethylene isosorbide terephthalate) (PICT) by electrospinning process were tested for potential wound dressing application. PICT/honey nanofibers with a 15% concentration of honey had the best mechanical properties, good releasing behavior and could, therefore, be used in wound dressing [81]. Similarly, electrospinning was used to fabricate zein nanofiber mats loaded with ethanol extracts of propolis. The scanning electron microscopy images revealed that with the increase in propolis content, the nanofiber size gradually increased along with the broadening of the size distribution probably due to a decrease in the ionic conductivity of the solutions [82].

Besides wound dressings, propolis was used to improve the antimicrobial properties of suture materials that are often the place of biofilm formation connected with surgical site infections. Baygar developed silk suture coated with propolis and biogenic silver nanoparticles (bioAgNPs) with wound healing properties. Biocompatible bioAgNP-propolis-coated sutures had potent antibacterial activity against *E. coli* and *S. aureus* and were able to simulate the proliferation of fibroblast [83]. Furthermore, propolis was used as an active ingredient in emulgel formulation developed for better management of wound and burn healing process [84]. Recently developed formulations for wound treatment based on bee products are summarized in Table 1. Most of them are prepared with propolis or honey to enhance the wound healing effect [81,82,83,84,85,86,87,88,89,90]. Prior to clinical evaluation of these wound-healing formulations, acceptability and efficacy must be assessed in comparison with conventional treatment options. Indeed, the most important steps in the development of wound care formulations are the evaluation of biocompatibility, antimicrobial activity and in vivo wound-healing activity [91]. Table 1 presents major newly developed formulations that are still to be fully assessed within pre-clinical studies. However, some of them, such as the bilayer wound dressing composed of a polyurethane/propolis membrane and a polycaprolactone/gelatin nanofibrous scaffold, already proved to be potential candidates for biomedical applications [87].

Encapsulation within polymeric matrix and development of nanoparticles was also tested with the aim to enhance low permeability and water solubility as well as to overcome the chemical instability of propolis that may affect its therapeutic properties [88,94]. Encouraging results for propolis-based nanoparticles were achieved in pathogenic biofilms destruction. Still, nanoformulations require pre-clinical and clinical studies on biological effects and particularly on safety and toxicology profiles. Recently, an appealing strategy in the delivery of bioactive compounds from bee products has been based on naturally occurring extracellular bee product vesicles.

## 4. Extracellular Vesicles as New Nutraceuticals from Bee Products

### 4.1. Extracellular Vesicles

Extracellular vesicles (EVs) are lipid bilayered vesicles released by both eukaryotic and procaryotic cells as extracellular messengers to mediate communication between cells [95]. Three major types of animal EVs are (1) exosomes with diameter ranges from 30 to 200 nm, (2) microvesicles with a diameter from 100 to 1000 nm and (3) apoptotic bodies with a diameter from 500 to 2000 nm. Exosomes are formed by an endosomal route, and microvesicles are released from the cell membrane, whereas apoptotic bodies are products of apoptotic cell death [96]. Recently, however, a more generalized nomenclature has been suggested for the classification of EVs based on the size and origin. Accordingly, categories of small extracellular vesicles (sEV, <100 nm), medium extracellular vesicles (mEV, <200 nm), large extracellular vesicles (L-EV, >200 nm) and exomeres (<35 nm) have been introduced [97].

**Figure 2:**
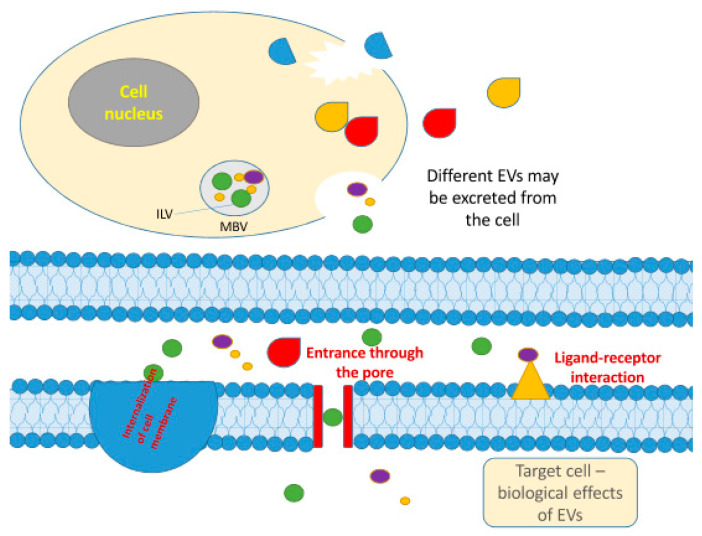
The generation for EVs within the cell and their role in cell-to-cell communication. Released EVs are heterogeneous and have different sizes. Among them, exosomes (up to 200 nm) are formed in the endolysosomal pathway as intraluminal vesicles (ILVs) within multivesicular bodies (MVBs). Different EVs may be formed and released from the cell. EVs affect the target cells through the delivery of their content into the target cell through internalization, ligand-receptor interaction or pore entrance.

EVs shuttle a set of molecules such as lipids, nucleic acids, proteins associated with the plasma membrane or cytosol and those related to lipid metabolism, nutrients and bioactive compounds [98,99]. EVs are produced within the plant or animal/human cell and are involved in cell-to-cell communication (Figure 2). Several methods were used to isolate EVs from biological fluids, including blood plasma, milk and urine. Among them, differential ultracentrifugation is the most widely used technique mainly because of its simplicity and cost-effectiveness [99,100,101]. Recently, EVs have become a subject of increasing interest due to the possible role even in communication between different species [95]. Researchers are now trying to understand if plant-derived extracellular vesicles can be absorbed in the mammalian gastrointestinal tract and be part of communication between different kingdoms [102]. Extracellular vesicles found in plants resemble exosomes in structure and function, but their biogenesis still remains elusive [99].

Exosomes are being studied in various therapeutic areas as they have shown many benefits. The administration of exosomes may be based on the therapeutic properties of the host, or the exosomes may be loaded with a therapeutic cargo [95]. Therefore, exosomes are being investigated as therapeutic agents, diagnostic targets or drug delivery vehicles [96]. EVs could be used as biomarkers in different medical settings, such as in the early and precise detection of several cancer types, diabetes, infectious and neurodegenerative diseases and autoimmune disorders. The successful application of EVs as therapeutic agents has been achieved, particularly in regenerative medicine [102]. For example, EVs from mesenchymal stem cells exerted anti-inflammatory and immunomodulatory activity associated with a positive influence on tissue regeneration after cardiac injury [103]. Additionally, EV preparations are tested as drug carriers in cancer therapy due to several benefits, including increased bioavailability and reduced toxicity of cytostatic agents [102]. However, in vivo distribution studies and prediction of therapeutic doses or potential side effects are required prior to the development of EV-based therapeutics and EV-based drug delivery vehicles for human use [96]. In addition, sustainable production or isolation of high yield exosome vesicles needs to be developed to fully exploit their competitive properties. [99].

### 4.2. Extracellular Vesicles in Bee Products and Their Effect on Wound Healing

Extracellular vesicles from different bee products have just recently attracted the attention of researchers [104]. Previous studies showed that exosomes from various animal sources are structurally similar to exosomes from human body fluids [95]. The study conducted by Schuch et al. (2019) has confirmed that *A. mellifera* hypopharyngeal gland secretomal products royal jelly, honey and bee pollen contain exosome-like vesicles (ELV) [104]. Examined ELV had different particle count profiles, i.e., honey and bee pollen displayed heterogeneous particle sizes, whereas royal jelly presented a uniform particle profile. Furthermore, the authors showed that protein-containing exosome-like vesicles participate in the antibacterial and pro-regenerative activity of bee-derived products. All exosome-like vesicles exerted bacteriostatic, bactericidal and biofilm-inhibiting capabilities on *S. aureus*, although ELV from royal jelly demonstrated the strongest activity toward the growth of bacteria in solution and biofilm formation on the surface. These results suggest that ELV from bee products could be used to prevent and treat wound infections during the healing process. Finally, the authors showed that the bee-derived vesicles are internalized by mesenchymal stem cells and that ELV can subsequently influence mammalian cell behaviour. Namely, ELV from honey and royal jelly significantly increased migration of mesenchymal stem cells what is thought to be one of the crucial steps in mammalian wound healing models to assist in immunomodulation, recruitment of fibroblasts and regeneration [105]. Recently, Chen et al. (2021) identified vesicle-like nanoparticles (VLN) in honey as new bioactive components [106]. The authors confirmed the presence of plant-originated plasma transmembrane proteins and plasma membrane-associated cytosolic proteins in VLN but failed to detect tetraspanins, a well-established EV marker. VLN from honey showed anti-inflammatory activity as they ameliorated NLR family pyrin domain containing 3 (NLRP3) inflammasome activity in primary macrophages. Screening of miRNAs revealed that miR-4057 from VLN suppressed NLRP3 inflammasome activation. In addition, the administration of VLN in mice reduced inflammation and liver damage.

**Figure 3:**
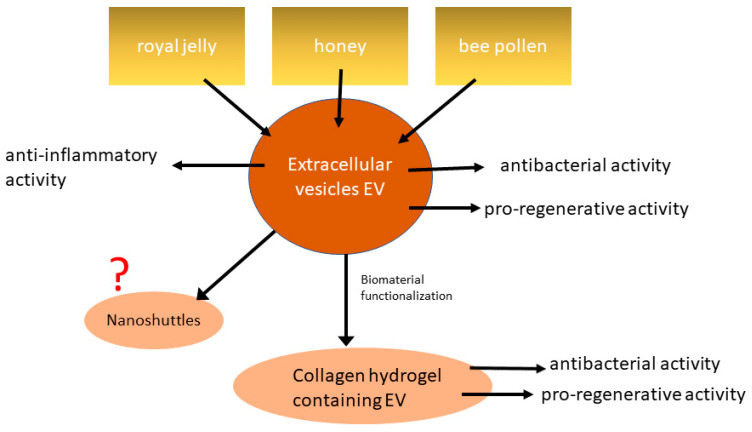
EVs have been identified in royal jelly, honey and bee pollen. Extracellular vesicles derived from bee products have antibacterial, pro-regenerative and anti-inflammatory activity that might be used in various medical applications.

Furthermore, the research group that first identified bee product-derived extracellular vesicles tried to develop novel type I collagen hydrogels as a continuous delivery matrix for royal jelly-derived extracellular vesicles [93]. Type I collagen hydrogels are well-established biomaterials for wound healing that can be also a local delivery system for EVs [107,108]. On the other hand, EVs have emerged as an interesting alternative in the clinical management of chronic wounds [109]. Therefore, the combination of EVs and type I collagen may offer a number of benefits in wound healing therapies. Ramírez et al. (2020) showed that the collagen concentration determines release patterns of royal jelly-derived extracellular vesicles and that gels containing 2 mg/mL collagen display the most stable release kinetics [93]. Released extracellular vesicles were biologically active as the integration into collagen did not alter their size or integrity. Functional EVs released over the time course of up to 7 days was demonstrated. Furthermore, collagen gels containing royal jelly-derived extracellular vesicles significantly reduced *S. aureus* ATCC 29213 biofilm formation compared to the control group. This study is an interesting example of targeted delivery of EVs from the bee-derived product using scaffolds. Figure 3 provides an overview of the current knowledge on EVs from bee products. So far, the role of EVs in wound healing has been confirmed, but the full potential of EVs and its medial application remain to be discovered.

### 4.3. Potential Perspective of EVs from Bee Products

Some of the first studies showing that bee product-derived extracellular vesicles may play an important role in the health-promoting activity of bee products. Further research is needed to confirm the findings as well as to investigate other possible applications of EVs from bee products. For example, bee product-derived extracellular vesicles could be used as nanoshuttles for different bioactive compounds (Figure 3). It was shown previously that exosomes could enhance the bioavailability of bioactive compounds due to their ability to increase their content stability. Exosomes can resist the enzymes during digestion as well as enzymes from other biological fluids, and therefore, their contents are protected from enzymatic degradation until they reach their target [99,110]. Animal-derived exosomes are stable at low temperatures, but their properties could be affected by different factors such as pressure and storage duration. To achieve the highest loading efficiency and improve the use of EVs for delivery, compounds naturally present in EVs may need to be removed so that bioactive compounds of interest can be loaded using two main methods: passive and active loading [99].

In addition to increased stability under digestive tract conditions, the application of EVs in delivery may have many other advantages. Exosomes are natural nanocarriers, so they are expected to be more biocompatible and safer than, for example, synthetic liposomes. They also have advantages in targeting, as their unique surface composition allows them to bind to specific targets [99]. In addition, exosomes can be engineered to further improve their targeting capability by adding proteins that target specific cells [111]. An interesting example of using animal exosomes to deliver bioactive compounds was demonstrated by Vashisht et al. [112]. The authors used milk exosomes to encapsulate curcumin, which despite having numerous therapeutic properties, has limited use due to poor water solubility, low systemic bioavailability and stability. Curcumin-encapsulated milk exosomes were resistant to human digestion and showed enhanced intestinal permeability, confirming that milk exosomes can act as stable oral drug delivery vehicles.

Whether extracellular vesicles derived from bee products can also be used as delivery vehicles for bioactive compounds or drugs remains to be investigated. Requirements that will have to be met for such an application are an efficient extraction and isolation process and a high extraction yield.

## 5. Conclusions

Bee products are a rich source of valuable bioactive compounds. Each bee product has a characteristic chemical profile that determines its biological activity. Royal jelly is best known for the major royal jelly protein 1, also called royalactin, while the bioactive compounds from propolis and honey that receive the most focus in research are polyphenols. It should be kept in mind, however, that since bees have different nutritional behavior and collect nourishments from different plants, the same type of bee product may substantially vary in composition, thus having a different health-promoting efficacy depending on its final composition. For example, the chemical composition of propolis depends on the geographical region, season, time of collection, altitude and availability of food during propolis exploitation [63]. Therefore, further development of analytical technologies, especially HRMS, will contribute to the complete characterization of the bioactive composition in bee products and a better understanding of the link between chemical profile and biological activity. Although bee products have demonstrated various health benefits, there has been an increasing interest in their application in the area of wound healing. Indeed, scientific data continuously confirm the role of bee products in accelerating the healing process. Observed regenerative properties of bee products are based on combined antimicrobial, antioxidant, immunomodulatory and anti-inflammatory activities. Researchers are now trying to develop new formulations for wound healing with bee products as active agents. The first promising results have also been obtained with extracellular vesicles from bee products. However, it is clear that further studies are warranted to elucidate the role of bee products’ extracellular vesicles as novel nutraceuticals and their benefits in the wound healing process.

## Figures and Tables

**Table 1:** Newly developed formulations based on bee products to promote the wound healing process.

Bee Product	Type of Formulation	Other Active Substances	Biocompatibility	Antimicrobial Activity	Wound Healing Effect	Reference
Beetosan–chitosan obtained from naturally died honeybees bee pollen	chitosan-based hydrogels	caffeine, bee pollen, *Salvia officinalis* (sage) and *Aloe vera* juice	fibroblasts	NA	NA	[80]
honey	chitosan-based hydrogels	No	NA	*Pseudomonas aeruginosa Staphylococcus aureus* *Klebsiella pneumonia Streptococcus pyogenes*	Yes in vivo	[85]
honey	poly (vinyl alcohol) (PVA) hydrogel with borax as a crosslinking agent	No	fibroblasts	*Escherichia coli* *S.aureus*	Yes in vitro	[86]
honey	poly(1,4-cyclohexane dimethylene isosorbide treph-thalate) (PICT) nanofibers	No	NA	NA	NA	[81]
propolis	zein nanofiber mats	No	NA	*S. aureus* *Staphylococcus epidermidis* *Candida albicans*	NA	[82]
propolis	bilayer wound dressings: polycaprolactone/gelatin (PCL/Gel) scaffold and polyurethane membrane	No	fibroblast	*S. aureus* *E. coli* *S. epidermidis*	Yes in vivo	[87]
propolis	silk suture	biogenic silver na-noparticles (bio-AgNPs)	fibroblast	*E. coli* *S. aureus*	Yes in vitro	[83]
propolis	emulgel	No	NA	NA	Yes in vivo	[84]
propolis	chitosan-propolis nanoparticles	No	NA	*E. faecalis* biofilms	NA	[88]
bee pollen	ointment containing 95% of petroleum jelly	No	NA	*Staphylococcus hyicus* *P. aeruginosa*	Yes in vivo	[92]
propolis propolis by-product	solid lipid nanoparticles (SLN) and nanostructured lipid carriers (NLC)	No	keratinocytes	NA	Yes in vivo	[89]
propolis	liposomes	No	NA	*Enterococcus faecalis* ATCC 29212 *S. aureus* ATCC 29213 *E. coli* ATCC 25922 *P. aeruginosa* ATCC 27853 *Candida albicans* ATCC 90028 *C. krusei* ATCC 6258 *C. parapsilosis* ATCC 90018	NA	[90]
royal jelly derived extracellular vesicles	type I collagen hydrogels	No	fibroblast	*S. aureus* ATCC 29213 biofilms	Yes in vitro	[93]

NA (not available).
